# Identification of cross-diagnostic biomarkers in ankylosing spondylitis and sarcopenia by bioinformatics and machine learning

**DOI:** 10.1097/MD.0000000000045916

**Published:** 2025-11-07

**Authors:** Yishaer Ka, Yingxin Liu, Lanqi Li, Hui Jiang, Wenbo Lv, Lei Wang, Xiaojin He

**Affiliations:** a The Affiliated Hospital of Nanjing University of Chinese Medicine, Nanjing, China; b Jiangsu Provincial Hospital of Traditional Chinese Medicine, Nanjing, China; c Nanjing Hospital of Chinese Medicine Affiliated to Nanjing University of Chinese Medicine, Nanjing, China.

**Keywords:** ankylosing spondylitis, bioinformatics, hub genes, machine learning, SARC

## Abstract

Ankylosing spondylitis (AS) and sarcopenia (SARC) often coexist, leading to impaired mobility through reduced muscle strength and altered bone metabolism. This study aimed to identify core diagnostic genes linking AS and SARC. This research analyzed 2 AS and 1 SARC dataset from the Gene Expression Omnibus database. Moreover, module genes and differentially expressed genes (DEGs) were evaluated *via* linear models for microarray data (Limma) and the weighted gene co-expression network analysis. Furthermore, functional enrichment analysis, various machine learning (ML) algorithms, and protein–protein interaction networks were employed for elucidating key candidate genes for the diagnosis of AS patients with SARC. The Receiver Operating Characteristic curve plots were utilized to determine the diagnostic significance of key genes. The merged AS dataset identified 1768 and 438 DEGs and module genes, respectively, in SARC. The intersection of module genes in SARC and DEGs in AS revealed 287 genes, which were predominantly enriched in oxidative phosphorylation. The protein–protein interaction network indicated 30 node genes. Furthermore, ML analysis identified 10 candidate hub genes for diagnostic value evaluation. In total, 6 candidate genes indicated high diagnostic significance key genes with the area under the curve > 0.7. The current study determined 6 hub genes (*ENSA, FAM43A, MDH2, NUBP1, SAMM50*, and *TM2D1*) for diagnosing AS patients with SARC, therefore providing a theoretical reference for potential diagnostic targets in these patients.

## 1. Introduction

Ankylosing spondylitis (AS) is a chronic inflammatory condition caused by immune-modulation and primarily affects the sacroiliac joints, spine, and peripheral joints.^[1]^ It has been indicated that AS is a type of axial spondyloarthritis (SpA), characterized by disease progression and deterioration,^[2]^ causing spinal deformity and even disability, which negatively impacts patients’ quality of life and imposes significant economic and social burdens on society. Sarcopenia (SARC) manifests with progressive and generalized muscle mass loss, reduced muscle function, and declined physical performance, which increases frailty, disability, and mortality.^[3,4]^ SARC was initially considered an age-related syndrome; however, later research revealed that disease-associated SARC might be associated with chronic inflammatory diseases and a lack of physical activity.^[5,6]^ In recent years, it was observed that AS patients have a substantially higher incidence of SARC than in the healthy population.^[7]^ In AS, the chronic inflammatory state is modulated by the secretion of pro-inflammatory cytokines like interleukin-6 (IL-6), tumor necrosis factor-α (TNF-α), and immune cell dysregulation.^[8]^ These inflammatory mediators can cause muscle atrophy and inhibit muscle regeneration, resulting in the onset of SARC,^[9]^ significantly impacting patients’ quality of life and overall health.

**Figure 1.**
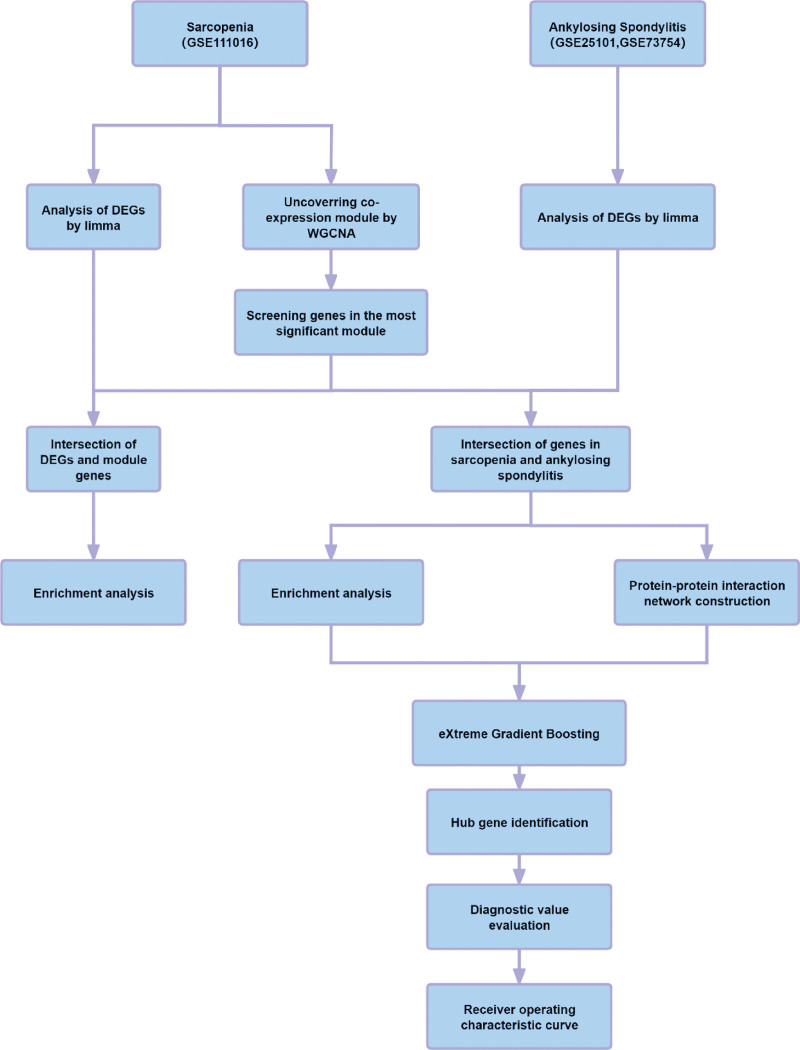
Illustration of the research design. DEG = differentially expressed genes, WGCNA = weighted gene co‑expression network analysis.

Bioinformatics analysis is a promising and efficient tool for identifying substantially aberrantly expressed genetic pathways and genes.^[10]^ Based on high-throughput sequencing technologies, integrated analysis techniques have been developed, which employ biological networks to provide a novel understanding of disease processes and identify biomarkers. This study employed genome-wide significance level instrumental variables to evaluate the bidirectional causal association between SARC and AS. The data revealed a potential bidirectional association between these 2 diseases (Fig. 1). Muscles and bones are formed from ectomesenchymal and mesodermal stem cells, respectively. These structures are anatomically proximate and modulate the exchange of chemical and mechanical signals. Identifying common shared regulatory genes may provide new avenues for prevention and treatment.^[11]^ After discovering common genetic markers, their genetic functions in animal or cellular models were elucidated, and their possible association with disease advancement was identified. Moreover, tissue or blood sample-based molecular diagnostic tests were developed to screen for the risk of AS and SARC. The findings will help develop novel gene therapies or drugs targeting the level or function of these genes.

Compared with previous studies that mainly focused on either rheumatologic disorders or metabolic diseases independently, the present work emphasizes the shared molecular mechanisms between AS and SARC. By integrating bioinformatics and machine learning, this study provides a systematic perspective on cross-diagnostic biomarkers, particularly highlighting mitochondrial energy metabolism as a potential convergent pathway. These insights may contribute to bridging basic molecular research with future strategies for individualized diagnosis and management in patients affected by both conditions.

## 2. Materials and methods

### 2.1. Data collection

This research was approved by the Ethics Committee of The Affiliated Hospital of Nanjing University of Chinese Medicine. The GSE25101, GSE73754, and GSE111016 datasets were acquired from the Gene Expression Omnibus (https://www.ncbi.nlm.nih.gov/geo/). The GPL6947 (Illumina HumanHT-12 V3.0 expression beadchip), GPL16791 (Illumina HiSeq 2500 for Homo sapiens), and GPL10558 (Illumina HumanHT-12 V4.0 expression beadchip) platforms were employed for the microarray analysis of the GSE25101, GSE73754, and GSE111016 datasets, respectively. Jerome Feige *et al* provided the GSE111016 dataset that had transcriptional responses associated with SARC. Whereas the GSE25101 and GSE73754 datasets, which focus on AS, were contributed by Gethin Thomas, Eric Gracey, and their respective collaborators.

### 2.2. Determination of differentially expressed genes (DEGs) between AS and SARC

The empirical Bayes method^[12]^ was employed to analyze the GSE25101 and GSE73754 datasets to mitigate batch effects. The expression matrices were extracted from the merged AS and SARC datasets. The genes and samples with > 50% missing values were removed. The R software package “impute” and function “impute.knn” were employed to identify missing values. For data completion, the number of neighbors was set to 10. Moreover, the GSE111016 dataset was subjected to log2 transformation. The average gene expression was also determined and presented by multiple probes. Finally, the linear models for microarray data (Limma) software package were employed for DEGs with *P < .05* and |log2 fold change| > 0.1 criteria. The threshold range was based on the research by Liu et al.^[13]^

### 2.3. Module gene selection and weighted gene co‑expression network analysis (WGCNA)

The gene-gene association was assessed *via* the WGCNA.^[14]^ According to the gene expression profiles, the top 25% most significant DEGs were selected for WGCNA. The R package, goodSamplesGenes, was utilized for the WGCNA to eliminate abnormal samples and genes. Moreover, WGCNA was performed to develop a scale-free co-expression network. Initially, the outliers were detected, and abnormal samples were removed *via* hierarchical clustering. Then, a scale-free network was built, and the soft threshold β = 20 (scale-free *R*^2^ = 0.891) was selected using the pickSoftThreshold function. Therefore, the adjacency matrix was established and transformed into the topological overlap matrix. Different degrees were employed to develop module colors and gene dendrogram. Moreover, the correlation between each module and the differential samples was also assessed.

### 2.4. Functional enrichment analysis

The gene ontology (GO)^[15]^ database provides computable and structured data on the activity of genes and their products. The Kyoto Encyclopedia of Genes and Genomes (KEGG)^[16]^ database is employed to study gene systems. The enrichment analyses, including KEGG and GO, were conducted *via* the following R packages: stringi, ggplot2, colorspace, clusterProfiler, DOSE, org.Hs.eg.db, pathview, and limma. The thresholds were established at a *P*-value as well as a *q*-value cutoff of 0.05. The GO functional test comprises 3 primary types: biological process (BP), cellular component (CC), and molecular function (MF). For visualization, the highest 10 entries with the smallest *P*-values (*P < .05*) were considered. The circle’s size or the line’s length indicates the total genes enriched in GO/KEGG, while the color represents the importance of enrichment. KEGG and GO tests were carried out according to the overlapping DEGs in SARC with significant module genes in AS, and vice versa.

### 2.5. Construction of protein–protein interaction (PPI) network

The target dataset was downloaded into the STRING database (https://string-db.org/).^[17]^ The minimum interaction score was 0.4 and the species were Homo sapiens. Cytoscape version 3.10.2 was employed for network visualization. According to the degree value, the color depth, text size, and node size were adjusted. A higher degree value indicated darker colors, larger text, and larger node sizes, suggesting the node’s increased significance within the PPI network. The CytoNCA plugin was utilized for the Network topology assessment.

### 2.6. Machine learning (ML)

Candidate genes for diagnosing SARC and AS were identified by 4 ML algorithms. The R software package “caret” was employed to build ML models, which included Random Forest,^[18]^ support vector machine,^[19]^ generalized linear model,^[20]^ and eXtreme Gradient Boosting (XGB).^[21]^ The validity of the model construction was assessed *via* the residuals and receiver operating characteristic (ROC) curves, which identified the top 10 variables as potential candidate genes.

### 2.7. Diagnostic value evaluation

The ROC curve analysis was constructed for each hub gene identified by ML to assess its diagnostic value for AS and SARC. Genes with an area under the curve (AUC) > 0.7 were considered to have significant diagnostic value.^[22]^

## 3. Results

### 3.1. Identification of DEG

**Figure 2.**
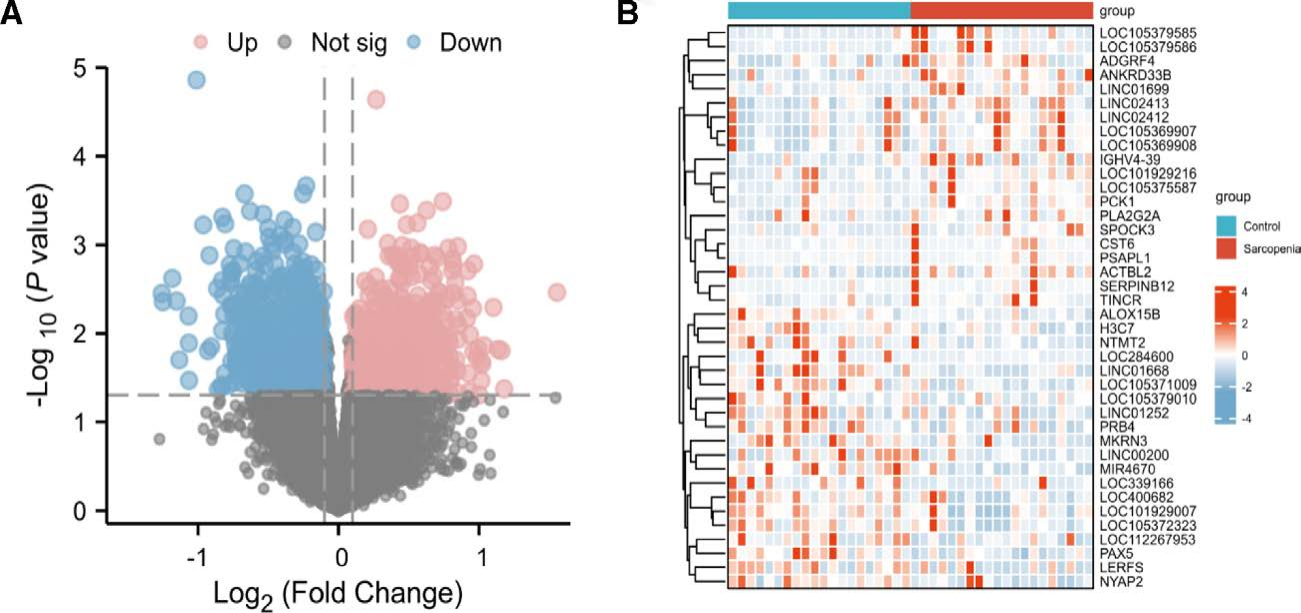
The volcano plot and heatmap of the DEGs in the SARC dataset. DEG = differentially expressed genes, SARC = sarcopenia.

**Figure 3.**
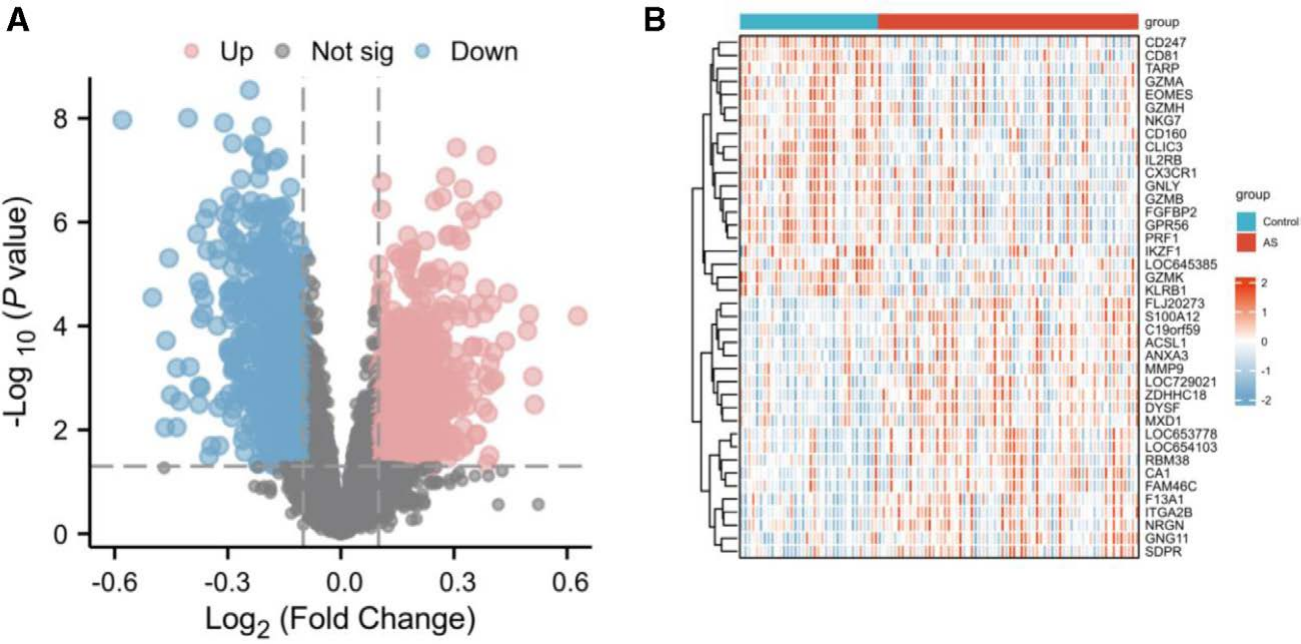
The volcano plot and heatmap of the DEGs in the AS dataset. AS = ankylosing spondylitis, DEG = differentially expressed genes.

The Limma method identified 1930 DEGs (1092 downregulated and 838 upregulated) in the SARC dataset. The volcano plot and heatmap of the DEGs in SARC are presented in Figure 2A–B. In the combined AS dataset, 1768 DEGs (844 upregulated and 924 downregulated) were identified. Figure 3A–B demonstrates the volcano plot and heatmap of AS-related DEGs.

### 3.2. WGCNA analysis and identification of key modules

**Figure 4.**
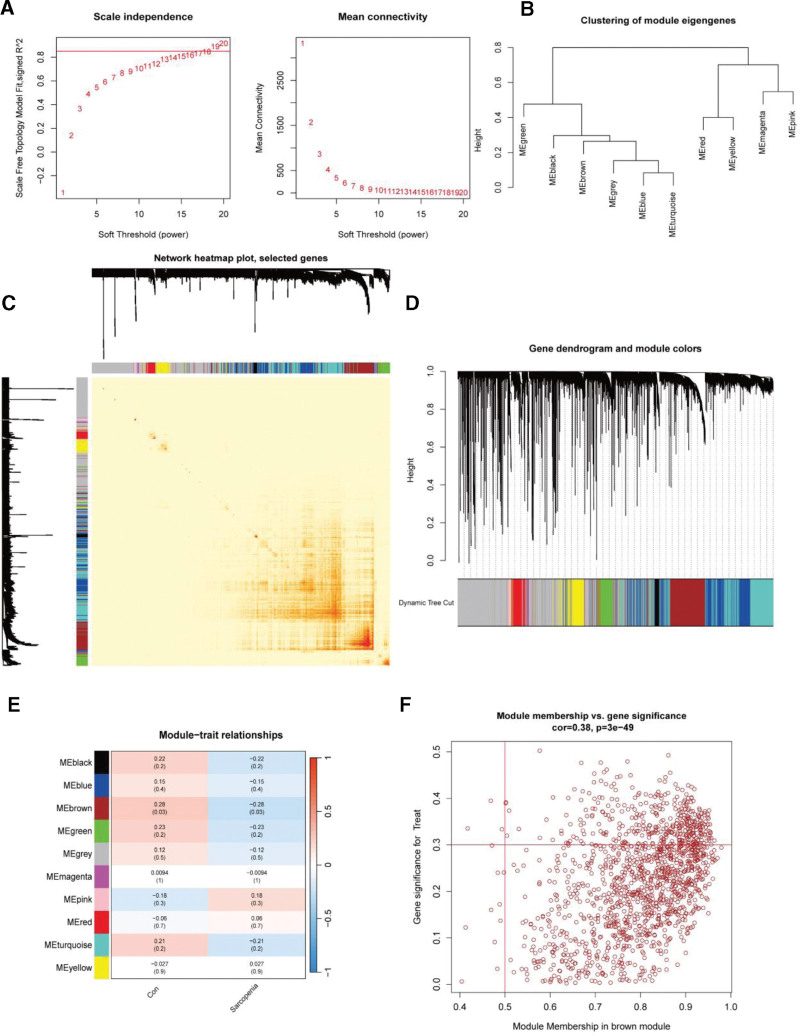
WGCNA of DEGs. (A) Network topology analysis across varying soft-thresholding powers. (B) Heatmap indicating the topological overlap within the gene network. (C) The association analysis between all identified modules. (D) Dendrogram of gene clustering, derived from hierarchical clustering based on adjacency dissimilarity. (E) Each row represents a module eigengene, while each column corresponds to a clinical phenotype. Each cell displays the correlation in the first row and the *P-value* in the second row, color-coded based on the correlation per the color legend. (F) The scatter plot indicates a highly significant correlation between gene significance (GS) and module membership (MM) in the brown module associated with SARC. DEG = differentially expressed genes, SARC = sarcopenia, WGCNA = weighted gene co‑expression network analysis.

Hierarchical clustering was performed to identify outliers and eliminate abnormal samples. Furthermore, a scale-free network was built using the function pickSoftThreshold, with a soft threshold of β = 20 (scale-free *R* = 0.916) (Fig. 4A). The correlation coefficient *R*² of > 0.8 indicated that this soft threshold was appropriate for establishing a scale-free network. Moreover, the adjacency and correlation matrices of the gene expression profiles were measured to construct a topological overlap matrix. Figure 4B illustrates the gene cluster tree. The data were converted into topological overlap matrix *via* the hierarchical average linkage clustering approach (Fig. 4C). The dynamic tree cut algorithm was employed for determining 10 gene modules (Fig. 4D). The gene-module correlation heatmap revealed a significant negative correlation coefficient of 0.28 between MEbrown and SARC samples (*P < .05*) (Fig. 4E). Based on gene importance and gene-module correlation, genes within each module were filtered, with filtering thresholds set at geneSigFilter = 0.3 and moduleSigFilter = 0.5. In total, 638 genes in MEbrown met the criteria (Fig. 4F).

### 3.3. SARC functional enrichment assay

**Figure 5.**
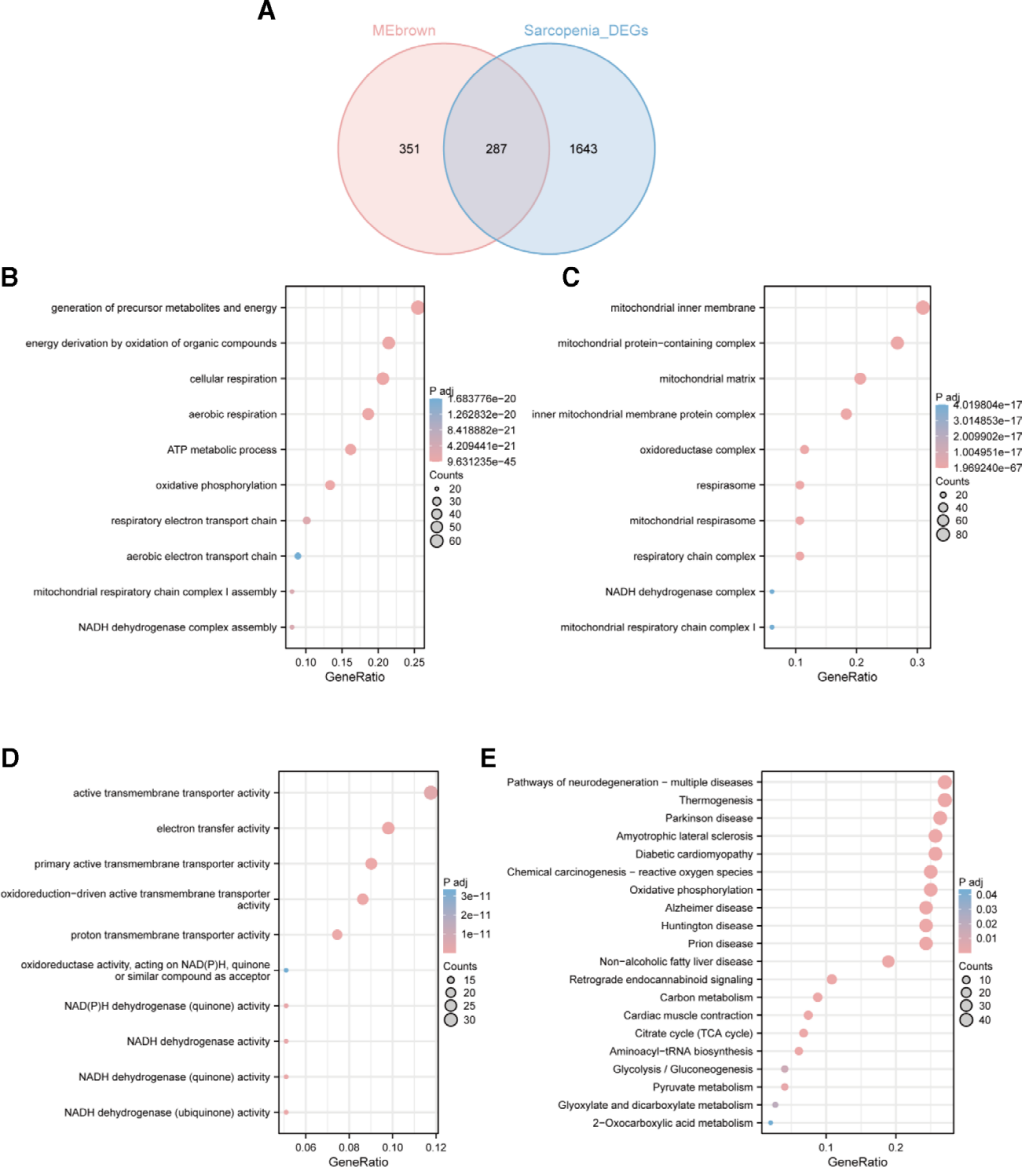
Enrichment analysis of intersecting genes in SARC. (A) The Venn diagram showed 287 intersection genes among the DEGs and the Brown module identified by the Limma method and WGCNA, respectively. Enrichment analyses of (B) Biological process, (C) Cellular component, and (D) Molecular function. (E) KEGG enrichment analysis. DEG = differentially expressed genes, KEGG = Kyoto Encyclopedia of Genes and Genomes, SARC = sarcopenia, WGCNA = weighted gene co‑expression network analysis.

To evaluate if the GSE111016 dataset reliably indicates SARC pathogenesis, a functional enrichment study was conducted using the intersected WGCNA and Limma module genes. The data identified 638 intersected genes from the MEbrown module with 1930 DEGs and 287 common genes (Fig. 5A). The GO and KEGG enrichment analyses of these 287 intersecting genes identified 114 BP, 53 CC, 35 MF, and 22 pathways. The significant BP were aerobic respiration, generation of energy, cellular respiration, and precursor metabolites, oxidative phosphorylation, and energy production by organic compounds (Fig. 5B). The important CC included the mitochondrial inner membrane, mitochondrial matrix, inner mitochondrial membrane protein complex, mitochondrial protein-containing complex, and respiratory chain complex (Fig. 5C). The essential MF were electron transfer, proton transmembrane transport, oxidoreduction-mediated active transmembrane transport, primary active transmembrane transport, and NADH dehydrogenase (ubiquinone) activity (Fig. 5D). The significant pathways included neurodegeneration-multiple diseases pathway, Amyotrophic Lateral Sclerosis, Huntington disease, nonalcoholic fatty liver disease, nonalcoholic fatty liver disease, Prion disease, diabetic cardiomyopathy, chemical carcinogenesis-reactive oxygen species (ROS), Parkinson disease, thermogenesis, and oxidative phosphorylation (Fig. 5E).

**Figure 6.**
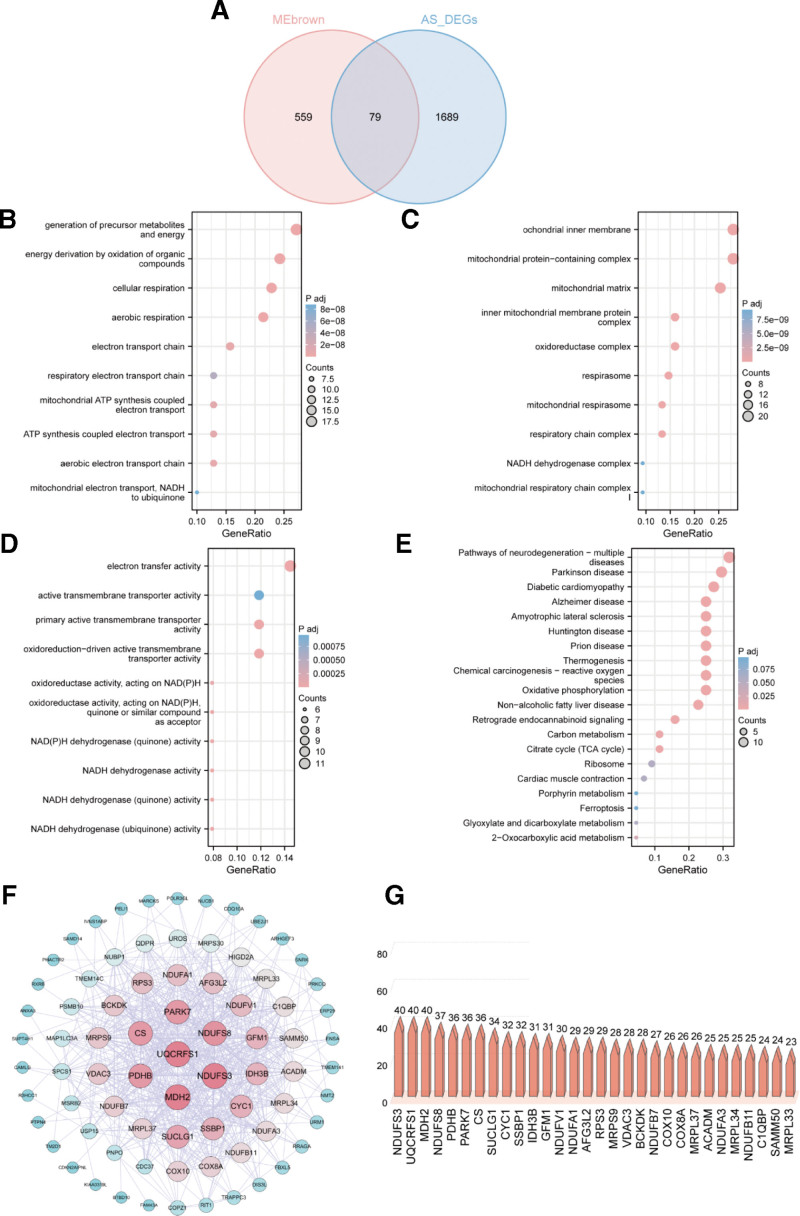
Enrichment test of common genes from AS with SARC and PPI network node genes analysis. (A) The Venn diagram represents the 79 common genes determined by the intersecting AS and SARC-related genes analyzed by the Limma and the WGCNA method, respectively. (B–D) GO analysis (including BP, CC, and MF) was performed on the 79 common genes. (E) KEGG pathway examination was conducted on the 79 common genes. (F) PPI network. (G) The bar chart displays the gene nodes of 30 genes in the PPI network. AS = ankylosing spondylitis, BP = biological process, CC = cellular component, KEGG = Kyoto Encyclopedia of Genes and Genomes, PPI = protein–protein interaction, SARC = sarcopenia, WGCNA = weighted gene co‑expression network analysis.

To elucidate whether the key genes in SARC are also associated with AS pathogenesis, the intersection of Brown module genes in SARC with DEGs in AS was visualized using a Venn diagram, which identified 79 genes (Fig. 6A). The GO enrichment analysis indicated the enrichment of 34 BP, 28 CCs, and 16 MFs. SARC may promote AS pathogenesis through various BPs, such as cellular respiration, energy production by organic compounds’ oxidation, energy generation and precursor metabolites production, aerobic respiration, and the aerobic electron transport chain (Fig. 6B); CC such as mitochondrial protein-containing complexes, mitochondrial inner membranes, mitochondrial matrices, oxidoreductase complexes, and respirasomes (Fig. 6C); as well as MFs including primary active transmembrane transporter activity, electron transfer activity, NADH dehydrogenase (ubiquinone) activity, NADH dehydrogenase (quinone) activity and oxidoreduction-driven active transmembrane transporter activity (Fig. 6D). The KEGG enrichment assay showed 20 pathways that were predominantly associated with the pathogenesis of SARC and AS. These pathways included diabetic cardiomyopathy, Parkinson disease, oxidative phosphorylation, chemical carcinogenesis related to ROS, nonalcoholic fatty liver condition, and thermogenesis (Fig. 6E).

After filtered genes validation, the PPI network was built to determine the interacting node genes for further ML screening. Figure 6F shows the PPI network comprising 77 nodes and 616 interactions. These genes are ranked according to their node degree, and the top-ranked genes included *NDUFS3, UQCRFS1, MDH2, NDUFS8, PDHB*, and *PARK7* (Fig. 6G).

### 3.4. Identification of candidate hub genes *via* ML

**Figure 7.**
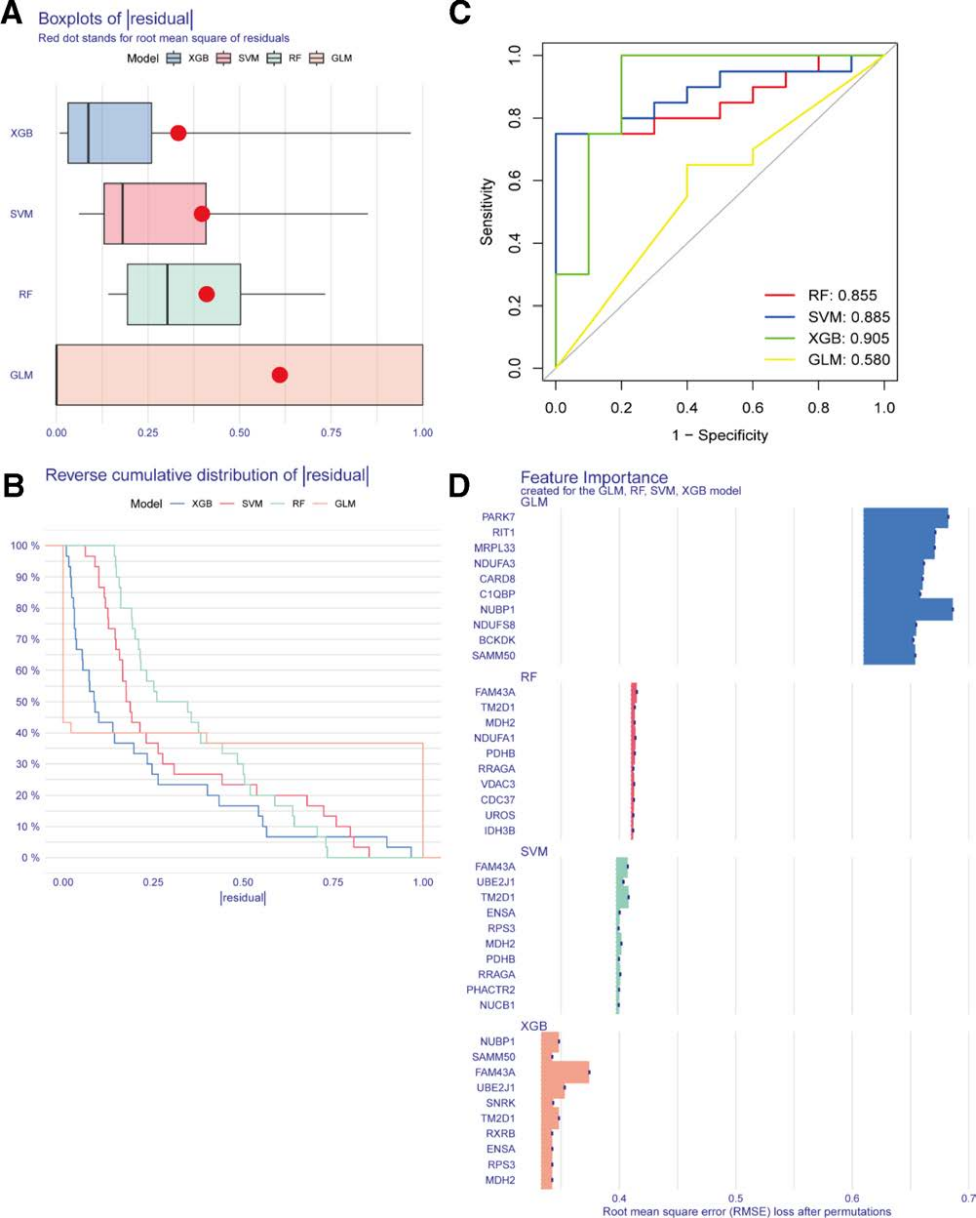
Machine learning was employed for identifying candidate diagnostic bio-indices for AS with SARC. (A) Box plot of residuals for ML model evaluation. (B) Waterfall plot of residuals for ML model evaluation. (C) ROC analysis for ML model evaluation. (D) Hub genes identified by 4 ML methods. AS = ankylosing spondylitis, ML = machine learning, ROC = receiver operating characteristic, SARC = sarcopenia.

The ML model was established according to the expression matrix of 79 genes *via* the R software package “caret.” The model’s rationality was evaluated by residuals and ROC analyses. The findings showed that the XGB ML method’s residuals were relatively small (Fig. 7A–B). Furthermore, the ROC evaluation of these 4 ML methods indicated that the AUC values for XGB, Random Forest, and support vector machine were all > 0.7, with the ROC for XGB reaching 0.905 (Fig. 7C). Based on the residuals and ROC evaluation, the XGB model was selected as the optimal ML model for the current research work. The top 10 candidate hub genes were *RXRB, MDH2, RPS3, SAMM50, ENSA, SNRK, TM2D1, NUBP1, UBE2J1*, and *FAM43A* (Fig. 7D).

### 3.5. Diagnostic significance

**Figure 8.**
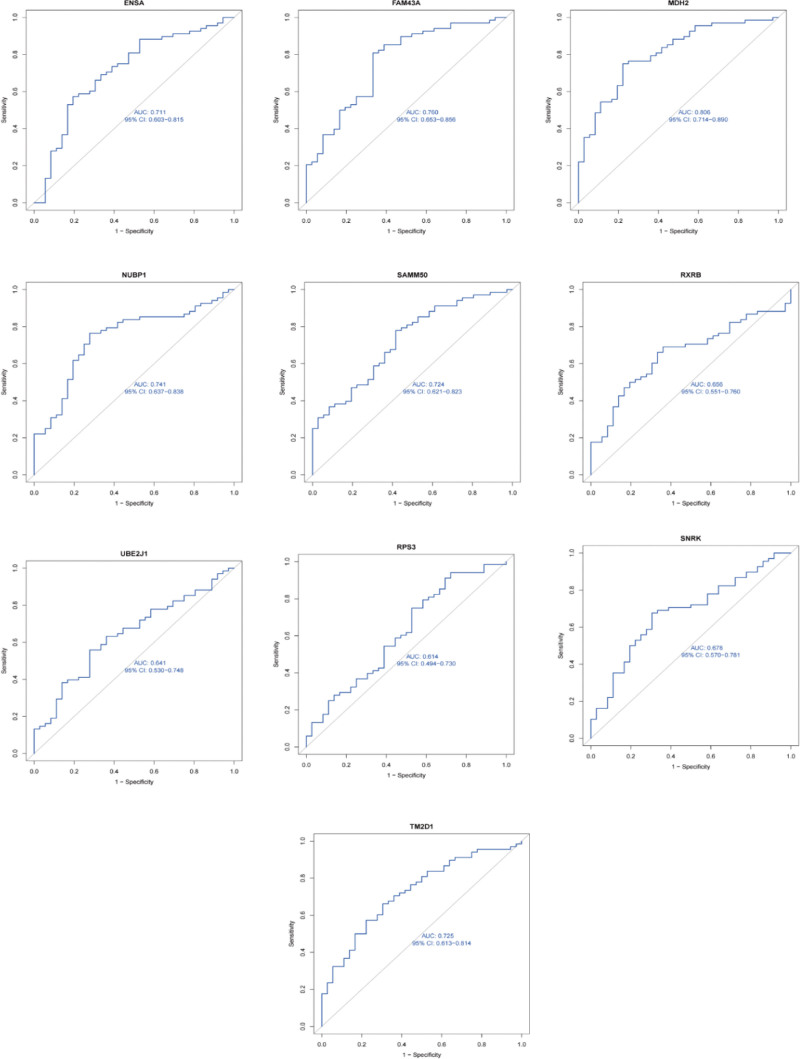
The evaluation of diagnostic values (The ROC curve of 10 candidate genes). ROC = receiver operating characteristic.

The diagnostic specificity and sensitivity of each gene were assessed by plotting the ROC curves of 10 hub genes (Fig. 8). The AUC for each item and its 95% confidence interval (CI) were identified. The results showed that AUC values >0.7 were observed for ENSA (CI: 0.603–0.815; AUC: 0.71), FAM43A (CI: 0.653–0.856; AUC: 0.76), MDH2 (CI: 0.714–0.890; AUC: 0.808), NUBP1 (CI: 0.637–0.838; AUC: 0.74), SAMM50 (CI: 0.621–0.823; AUC: 0.72), and TM2D1 (CI: 0.613–0.814; AUC: 0.72). These 6 candidate genes demonstrated good diagnostic value for AS combined with SARC.

## 4. Discussion

AS is an inflammatory disease classified under seronegative SpA. Its pathogenesis remains incompletely understood, and the underlying pathways are modulated by various factors. However, its primary etiology includes an autoimmune response-mediated inflammatory process.^[23]^ Common early symptoms include inflammatory back pain and characteristic stiffness that worsens with inactivity.^[24]^ Furthermore, it can also affect peripheral joints, which causes symptoms including synovitis, enthesitis, and dactylitis.^[25]^ Moreover, AS has been associated with several extra-articular manifestations, like inflammatory bowel disorder, psoriasis, and iritis.^[26]^

SARC manifests as the accelerated loss of skeletal muscle function and mass and has been, but not exclusively, related to aging.^[27]^ Recent studies have revealed that SARC is correlated with immune-mediated inflammatory diseases, such as AS. Researchers have identified that persistent chronic inflammation is a significant risk factor for SARC onset.^[28]^ Furthermore, the incidence of SARC is high in AS patients,^[29]^ which suggests the need for targeted prevention and treatment strategies for SARC in conjunction with managing AS.

Recently, the progress in sequencing technologies has increased the integration of bioinformatics analysis and ML methods to identify core genes related to diseases, potential diagnostic and prognostic biomarkers, in addition to signaling pathways and therapeutic marks. This integration provides substantial data support for the comprehensive assessment of diseases.^[30,31]^ However, the identification of biomarkers for AS with SARC using bioinformatics and ML algorithms has not been performed yet. Therefore, this study employed various comprehensive bioinformatics tests with multiple ML methods to identify hub genes for AS with SARC. Furthermore, the identified genes’ diagnostic significance for this condition was also evaluated. In total, 6 key genes (*ENSA, FAM43A, MDH2, NUBP1, SAMM50*, and *TM2D1*) with significant diagnostic potential were identified.

ENSA, also known as Alpha-endosulfine, is a crucial phosphatase inhibitor that specifically regulates protein phosphatase 2A during mitosis by inhibiting its activity. The literature has indicated that ENSA expression is significantly increased in triple-negative breast cancer, where it activates signal transducer and activator of transcription 3 (STAT3) to regulate cholesterol biosynthesis, thus promoting tumor progression.^[32]^ STAT3 acts as a convergence point for various oncogenic signaling pathways and has been linked with AS. It has been validated that STAT3 promotes the activation, propagation, and differentiation of Th1 cells and NKT cells, as well as in heterotopic ossification associated with AS.^[33]^ Endurance training has been found to alleviate SARC through the Janus kinase 2/STAT3 pathway.^[34]^ FAM43A is a gene that encodes a protein that can predict the prognosis of triple-negative breast cancer^[35]^ and is upregulated in membranous nephropathy.^[36]^ It was also observed that FAM43A expression is significantly increased during sepsis, leading to inflammatory responses and immune dysregulation.^[37]^ However, the role of FAM43A in AS and SARC remains elusive. MDH2 encodes mitochondrial malate dehydrogenase 2, which is substantially expressed in the heart and skeletal muscles. MDH2 dysfunction negatively affects the energy metabolism of muscle cells.^[38]^ Furthermore, MDH2 is crucially involved in the tricarboxylic acid cycle by converting malate into oxaloacetate, thus influencing the NADH/NAD + ratio and regulating nicotinamide adenine dinucleotide (NADH) levels.^[39]^ Increased NADH accumulation can induce cellular reductive stress, forming an immunosuppressive microenvironment that stimulates immunosuppressive cells’ differentiation and exacerbates tumor metastasis to immunosuppressive target organs.^[40]^ Recent data indicate that follicle-stimulating hormone also influences osteoclasts’ energy metabolism by activating the CREB-MDH2-NAD + signaling axis through its receptor, thus exacerbating bone loss.^[41]^ This is consistent with the present study, which suggests that AS patients have abnormal bone metabolism, including the incidence of osteoporosis.^[42]^ The NUBP1 gene encodes a protein of the NUBP/multidrug resistance-associated protein subfamily of ATP-binding proteins.^[43,44]^ NUBP1 is associated with metabolism and cytoplasmic iron-sulfur cluster assembly pathways. Furthermore, it crucially modulates centrosome replication and the assembly of cytoplasmic proteins.^[45,46]^ NUBP1 dysfunction can cause mitochondrial dysfunction, and accumulating ROS, which induces oxidative stress, triggering lipid peroxidation and deoxyribonucleic acid damage. This process activates the inflammasome in macrophages, which releases pro-inflammatory factors like TNF-α and IL-1β.^[47]^ Chronic inflammation is a fundamental characteristic of AS and a risk factor for SARC.^[6]^ Moreover, inflammatory cytokines, such as TNF-α, have been identified in AS and SARC, linking their development.^[48]^ SAMM50 is a protein in the mitochondrial outer membrane that is significantly involved in the clearance of ROS, mitochondrial morphology, and mitophagy regulation.^[49–51]^ Mitochondria are also crucial in inflammation and immune responses.^[52]^ Studies have reported that SAMM50 variants may reduce the production of Sam50, promoting mitochondrial dysfunction-mediated steatosis, which induces the development and progression of nonalcoholic fatty liver disease.^[53]^ The β-amyloid peptide-binding protein, TM2D1, has been associated with β-amyloid, the most recognized pathogenic factor in Alzheimer disease.^[54]^ Furthermore, TM2D1 also acts as a novel oncogene in hepatocellular carcinoma, related to enhanced proliferation and invasion capabilities.^[55]^ Further, TM2D1 regulates ferroptosis, and its inhibition increases the levels of cellular ROS.^[56]^ An abnormal elevation in cellular ROS frequently results in inflammation.

In addition, the cross-species applicability and translational feasibility of our findings warrant careful consideration. Although the hub genes identified here were derived from human transcriptomic datasets, their functional conservation across species remains to be established. Future studies in animal models of ankylosing spondylitis and sarcopenia will be essential to validate whether these genes play comparable mechanistic roles in vivo. From a clinical perspective, the potential of ENSA, FAM43A, MDH2, NUBP1, SAMM50, and TM2D1 as biomarkers is promising, as they may be measurable in peripheral blood or muscle biopsy specimens to support early screening and individualized diagnosis of AS patients at risk of sarcopenia. However, several obstacles remain before clinical translation. This investigation leveraged integrated bioinformatics and multiple machine‑learning methods to nominate candidate hub genes for diagnosing ankylosing spondylitis complicated by sarcopenia. Nonetheless, several limitations should be emphasized. First, the overall sample size was modest, and we did not perform external validation in independent public cohorts or in clinical specimens; therefore, the universality and reliability of the identified hub genes and the derived models may be limited. Second, the exclusive use of publicly available datasets and the cross‑sectional design may introduce selection and information biases; residual batch/platform effects cannot be completely excluded despite harmonization. Third, the present discovery‑stage results may not generalize to diverse populations or clinical settings. Future work will address these gaps through independent external validation using additional public datasets and multi‑center, adequately powered cohorts and experimental verification (e.g., qRT‑PCR/Western blotting in patient tissues or blood, and functional assays in cell/animal models). Collectively, our findings provide a basis for diagnostic index development, but further validation and clinical translation are required before these genes can be adopted in practice.

## 5. Conclusion

In summary, this research study systematically determined 6 candidate hub genes (*ENSA, FAM43A, MDH2, NUBP1, SAMM50*, and *TM2D1*) with promising diagnostic value. The results provide insights into prospective identifying genes for AS patients with SARC.

## Author contributions

**Conceptualization:** Yishaer Ka, Lanqi Li.

**Data curation:** Yishaer Ka, Yingxin Liu, Lanqi Li, Hui Jiang, Lei Wang.

**Formal analysis:** Yishaer Ka, Yingxin Liu, Lanqi Li, Hui Jiang, Wenbo Lv, Lei Wang.

**Investigation:** Yishaer Ka, Yingxin Liu, Lanqi Li.

**Methodology:** Yishaer Ka, Yingxin Liu, Lanqi Li.

**Validation:** Wenbo Lv.

**Writing – original draft:** Yishaer Ka, Yingxin Liu, Lanqi Li, Xiaojin He.

**Writing – review & editing:** Yishaer Ka, Yingxin Liu, Lanqi Li, Xiaojin He.
